# Facemask wearing does not impact neuro-electrical brain activity

**DOI:** 10.1038/s41598-022-12875-1

**Published:** 2022-05-31

**Authors:** Ahmad Tamimi, Said Dahbour, Assma Al-Btush, Abdelkarim Al-Qudah, Amira Masri, Subhi Al-Ghanem, Malik E. Juweid, Yazan Olaimat, Amer Al Qaisi, Qutada Al-Soub, Maha Naim, Ali Sawalmeh, Rund Jarrar, Tala Tarawneh, Mai Bader, Iskandar Tamimi

**Affiliations:** 1 Department of Neurosurgery, Jordan University Hospital and Faculty of Medicine, University of Jordan, Queen Rania Street, Amman, Jordan; 2 Department of Neurology, Faculty of Medicine, University of Jordan, Amman, Jordan; 3 Department of Respiratory, Faculty of Medicine, University of Jordan, Amman, Jordan; 4 Department of Pediatric Neurology, Faculty of Medicine, University of Jordan Hospital, Amman, Jordan; 5 Department of Anesthesiology, Faculty of Medicine, University of Jordan Hospital, Amman, Jordan; 6 Department of Radiology and Nuclear Medicine, Faculty of Medicine, University of Jordan Hospital, Amman, Jordan; 7 Department of Orthopedic Surgery, Hospital Regional Universitario de Malaga, Málaga, Spain; 8 Faculty of Medicine, University of Malaga, Málaga, Spain

**Keywords:** Neuroscience, Physiology, Health care, Medical research, Neurology

## Abstract

Given the massive use of facemasks (FMs) during the covid-19 pandemic, concerns have been raised regarding the effect of FMs wearing on overall health. This study aimed at evaluating the effect of surgical FM on brain neuro-electrical activity. Electroencephalography (EEG) background frequency (BGF) and background amplitude (BGA) was performed on 30 volunteers before (baseline), during and after wearing a FM for 60 min. Measurements were done during normal ventilation, hyperventilation and post-hyperventilation (PHVR). Blood gas levels were assessed at baseline and after FM use. EEG analysis concerning baseline (without FM) (BGA), was 47.69 ± 18.60 µV, wearing FM, BGA was 48.45 ± 17.79 µV, post FM use BGA was 48.08 ± 18.30 µV. There were no statistically significant differences between baseline BGA and BGA under FM and post FM. BGF, Baseline data were 10.27 ± 0.79, FM use 10.30 ± 0.76 and post FM use was 10.33 ± 0.76. There were no statistically significant differences between baseline BGF and BGF under FM and post FM. Venous blood gases, and peripheral oxygen saturation were not significantly affected by FM use. Short-term use of FM in young healthy individuals has no significant alteration impact on brain's neuro-electrical activity

## Introduction

On December 2019 the People's Republic of China (PRC) reported the first case of severe acute respiratory syndrome (SARS) associated with a novel coronavirus disease 2019 (COVID-19). This new virus was first identified in the city of Wuhan in the Hubei province^1^. Since then, it has spread to over 203 countries, and was officially declared as a global pandemic illness by the World Health Organization (WHO)^2^. After almost 2 years, the COVID-19 pandemic has left a toll of 446 million infected and more than 6.0 million deaths worldwide^3^.

From the beginning of the COVID-19 pandemic, the use of facemasks (FMs) has been controversial due to political, cultural and economic causes. However, multiple studies have highlighted the efficiency of FM in the reduction of COVID-19 transmission (i.e., from 6 to 80%)^4^. In another report the use of medical/surgical masks reduced the transmission rates by 85%^5^. Countries worldwide have adapted many different approaches to control the disease^6^. Non-pharmaceutical actions included the use gowns, gloves, and FM, all of which have been proven to be effective and more recently, the use of vaccines such as the BNT162b2 mRNA Covid-19 vaccine, Curevac, Moderna, AstraZeneca, and Sino pharm has proven effective in the control of the pandemic^7–10^.

Some side effects associated with the use of FMs have been reported. The most common side effect described by healthcare workers was bilateral headaches (affecting up to 80% of users), dermatitis, itchy rash and elevated heart rates^11^. However, the WHO has advised populations to wear FMs on a large scale only excluding patients with limited respiratory conditions^4^. Recently updated guidelines issued by the WHO advised the use of FMs as part of its prevention recommendations which also included hand hygiene, physical distance of at least 1 m, avoidance of face touching and adequate indoor ventilation^2,12^.

Previous clinical studies have addressed the potential impact of FMs on brain and cognitive functions^13,14^. One study in healthy young people showed that the 150-min use of surgical mask had no significant mental fatigue perception^13^ while another showed that the prolonged use of FMs can result in headache and impaired cognition^14^.

Recent physiological studies with capnogrphy while wearing FMs, demonstrated an increased end tidal (ET) CO_2_ by 7.4% causing increased cerebral blood flow(CBF) and inducing global gray matter activation^15^. Another study on the cerebral dynamic conditions of the brain and oxygenation, demonstrated small but significant changes in the cerebral hemodynamics while wearing a FM in young adults. However, the changes were similar to those of daily life activity and did not suggest a hypoxic effect^16^. Changes in cerebral blood flow (CBF) can affect brain metabolism and EEG activity^17^.

There is paucity of information on physiological effects of FMs on the bioelectrical activity of the brain. The current global use of FMs as a results of the current pandemic has motivated us to investigate the short-term impact of FMs use on the brain's neuro-electrical activity. This impact may be caused by the possible hypercapnia during inspiration while wearing FMs^18^. Our hypothesis is that the use of FMs could affect the bioelectrical activity of the brain due to possible hypercapnia or hypoxemia, before the development of clinical symptoms during hyperventilation and post hyperventilation during FM use or post FM use.

## Methods

### Study sample

We conducted a cross-sectional study performed on healthy volunteers to determine the impact of FM use on the brain’s bioelectrical activity and respiratory function. We included young and healthy subjects from a homogenous group of medical students. The following parameters were analyzed in all the participants: age, gender, length, weight, body mass index, heart rate, diastolic blood pressure, systolic blood pressure, and FM use time per day. The exclusion criteria were the presence of any respiratory or neurological illnesses.

All volunteers used a disposable surgical FM (HiTEX Manufacture of Medical Devices, Tybe IIR, NH0050, FDA CE, Amman, Jordan). The participants were investigated during 2 different sessions 72 h apart with all tests performed in the morning.

The study was carried out by the Neuroscience and Respiratory team at Jordan University Hospital (JUH) and was conducted in accordance with the Declaration of Helsinki and approved by our institutional review board committee (IRB) (reference number 10-2021-4251) at JUH. A written informed consent form was obtained from all participants and informed consent for publication of the image was obtained from the participant.

### Measures (day 1)

All demographic and general data were gathered at the beginning of the study, in day one.

### Respiratory function (spirometry)

We used VIASYS Health Care device (model Vmax Ecnone; S/N77400SEP05, GmbH, made in Germany) to determine the forced vital capacity (FVC), forced expiratory volume in one second (EFV1) and FEV1/FVC] ratio. The preparation of participants included the removal of tight clothes, and placement of a soft clip was on the nose with a sterile mouthpiece and filter attached to the spirometer.

For the respiratory procedures, participants took deep breaths, followed by a breath hold for a second, forced exhalation into the mouthpiece for 6 s, followed by another deep breath. The test was repeated three times to make sure that the results were consistent. The highest the reading was considered as the tests result. In spirometry, the FEV1/FVC was calculated from the FEV1 and FVC results. The device used the predicted values to provide FVC, EFV1 percentages and FEV1/FVC ratio for the test subjects.

### EEG measurement, acquisitions and analysis

**Figure 1:**
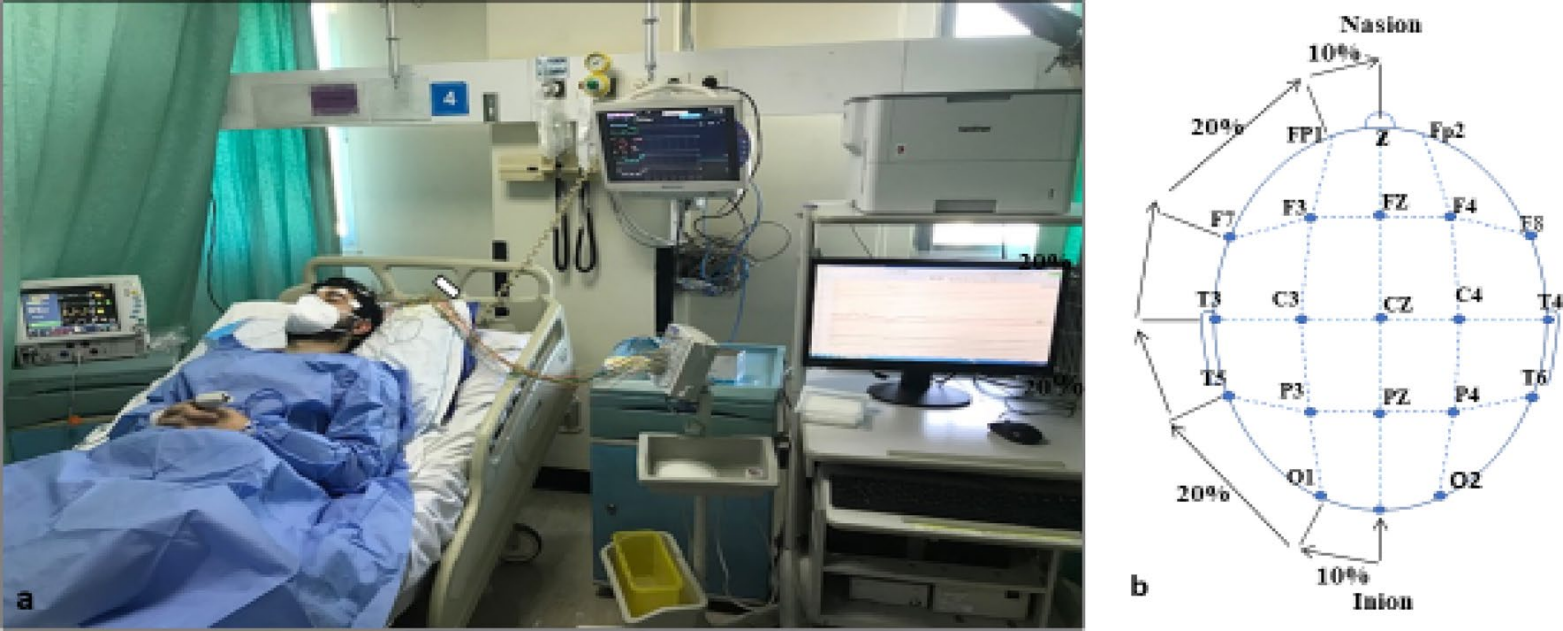
(**a**) Volunteer undergoing EEG recording and monitoring, with face mask; (**b**) Electrode placement diagram used in our study, 10–20 system.

Baseline phase, (without FMs): We used Nihon Kohden Electroencephalograph, model EEG 1200, female connector plugs 1.5 cm (Nihon Kohden Corporation, 1-31-4-Nishiochiai, Shinjuku, Toky0, 161-8560, Japan). There were 22 electrodes type H-526 Nihon Kohden (reusable Collodion EEG Cup Electrode of silver Ag arranged via an augmented 10–20) (NIHON KOHDEN India Pvt Ltd, India,) 20 for EEG, output of 16, and channels; two chest electrodes for electrocardiography (ECG) output channel, and respiratory frequency/minute) (Fig. 1b). We used anterior posterior montage "double banana", sensitivity 10µv for EEG, 50µv for ECG, time constant; 0.1 s, high cut frequency filter: 70 Hz. EEG measurements were done in the morning with the volunteers fasting after midnight. The technician scrubbed the spots that were measured with a special skin preparation gel for active electrodes (NuPrep), (Ten20 conductive electrode paste, Weaver and Company 565, Nuprep skin, Nucla way. Unite B, Aurora. Colorado. 80011, USA). A sticky adhesive gel was applied on the 20 electrodes (conductive electrode paste), and attached to the spots over the scalp. The technician asked the volunteers to lie on bed, close their eyes and after 3 min of recording, the technician asked the volunteer to do hyperventilation (HV) breathing deeply through the nose and breathing out through pursed lips for 3 min in room air with a respiratory rate of approximately 20 to 25 breaths per minute under monitoring. After pausing the hyperventilation state, the technician recorded post-hyperventilation for 4 min, under monitoring of the ECG, and pheripheral oxygen saturation level (trough finger puls oximeter (Sp O_2_), Respiratory rate, and SpO_2_ were recorded through. GE Datex Ohmeda Cardiocap 5 (SOMA TECH INTL, 166 Highland Park Dr. Bloomfield. CT 06002. 1.800. Get. Soma.) (Fig. 1a).

Background frequency (BGF) and background amplitude (BGA) in microvolts without facemask at Scalp surface (EEG) epochs each for 10 min, were recorded while the participant was relaxed with his eyes closed to induce alpha background activity. Each epoch included: 3 min of normal breathing, 3 min of hyperventilation and 4 min of post hyperventilation. All data were collected and analyzed statistically.

### Measures (day 2)

On this day, participants wore FMs and oxygen saturation was measured as follows: Starting in the morning after 60 min of FM use, 10-min EEG recording were performed while the participants were still wearing the FMs, and then after taking off the mask for another 10 min. Each epoch included: 3 min of normal breathing, 3 min of hyperventilation and 4 min of post-hyperventilation. The following EEG parameters were measured while wearing the FMs: BGF, BGA, response during hyperventilation (DHVR) and post-hyperventilation response (PHVR). BGF and BGA (normal alpha 8–13 Hz and normal amplitude (0.5 to 100 microvolts) are frequently measured in daily EEG practice (Fig. 1b). Boxplots of background frequency and amplitude in the three phases of the study; baseline, FMs wearing and post-FMs wearing recording were generated. These parameters were compared to each other looking for any changes related to FM use.

Bassline Venous Blood Gases (VBGs) were extracted before FM wearing (1st sample), including PH, CO_2_, CHO_3_, BE, SO_2_ and O_2_ levels. Overall, all volunteers wore the FM for 70 min (2nd sample), (VGs samples were extracted for from the antecubital veins at 8 a.m. (day2) (BD Prest, Becton, Gickinson and company Rodborough, Plymouth, PL6 7BP, United Kingdom) as a base line (1st sample) before FMs use and (2nd sample) was extracted immediately before FMs removal.

Peripheral oxygen saturation (SpO_2_), were measured during FMs use just before its removal (Fig. 1a).

### Statistical analysis

The statistical analysis was performed using statistical analysis system IBM SPSS Statistics 20 (SPSS Inc., Chicago, IL, USA). The distribution of continuous variables was analyzed using the Shapiro–Wilk test. Means were presented with their corresponding standard deviations. Differences between continuous independent variables were analyzed using Student’s T Test and Mann Whitney U Test depending on the distribution of the variables. Differences between dependent variables were analyzed using Student’s T test for paired samples, and Wilcoxon test depending on sample distribution. Differences between dependent dichotomous variables were analyzed using Cochran’s Q Test. P values were considered statistically significant if less than 0.05. Sample size was calculated using G*power 3.0.10 (Universität Kiel, Germany). A priory analysis was performed using a t-test for two dependent means, with an α-error probability of 0.05 and β-error of 80%.

### Ethics

Approved by our institutional review board committee (IRB) (reference number 10-2021-4251).

### Consent

Consent was signed by all research volunteers.

## Results

### Population data

Demographic and General data of Volunteers are shown in Table 1. Thirty medical students (median age: 23.7 ± 1.7 (22–25 y) were included [i.e., 12 (40%) males, and 18 (60%) females]. The mean height, weight, and body mass index of the participants was of 168.2 ± 9.5 cm, 69.5 ± 14.7 kg, and 24.3 ± 4 kg/m^2^; respectively. The average heart rate, diastolic blood pressure, diastolic blood pressure, respirator rate and FMs per/day were 87.3 ± 11.3/min, 81.6 ± 6.6 mmHg, 120.5/11.4 mmHg, 20 ± 2/min, and 4.4 ± 1.9 h, respectively. The size sample was determined following similar studies in the literature.

**Table 1:** Demographic and general data of volunteers.

Parameter	Mean ± SD
Age (y)	23.65 ± 1.65
**Gender (total)**	30 (100%)
Male	12 (40%)
Female	18 (60%)
Length (cm)	168.16 ± 9.51 cm
Weight(kg)	69.51 ± 14.67
Body mass index (kg/m^2^)	24.25 ± 3.9
Heart rate/min	87.26 ± 11.29
Diastolic blood pressure (mmHg)	81.56 ± 6.56
Systolic blood pressure (mmHg)	120.5 ± 11.37
Wearing FM/day (hour)	4.41 ± 1.83

SD, standard deviation.

### Respiratory function analysis

Baseline respiratory functional analyses were within the normal range, revealing an average FVC of 4.5 ± 0.7 L, FEV1 of 3.6 ± 0.6 L, and an FEV1/FVC ratio of 80.1 ± 0.66% (Table 2).

**Table 2:** Baseline respiratory function data (spirometry test).

Parameters	Mean ± SD
Respiratory rate/min	19.96 ± 1.97
FVC(L)	4.52 ± 0.74
FEV1(L)	3.62 ± 0.59
FEV1/FVC	80.08 ± 0.66%

FVC, forced vital capacity; FEV1, forced expiratory volume in one second; SD, standard deviation.

### Electroencephalography (EEG) results

The baseline BGF was of 10.27 ± 0.79, whereas BGF for FM use was10.30 ± 0.76, and post FM use was of 10.33 ± 0.76. There were no statistically significant difference between baseline, FM use and post FMs BGFs (P = 0.317) and (P = 0.157), respectively (Table 3). Boxplot of background frequency in the three phases of the study baseline, wearing FMs and post-FMs recording showing no significant difference.

**Table 3:** Electroencephalographic(EEG) findings in the three phases of the study (n = 30).

	Baseline	Facemask use	Post facemask
BGA (mean ± sd mv)	47.69 ± 18.60	48.45 ± 17.97 (p = 0.528)^a^	48.08 ± 18.30 (p = 0.807)^a^
BGF (Hz mean ± sd)	10.27 ± 0.79	10.30 ± 0.75 (p = 0.317)^a^	10.33 ± 0.76 (p = 0.157)^a^
Slowing DHVR n (%)	6 (20.0%)	3 (10.0) (p = 0.375)^b^	7(23.3) (p = 1.000)^b^
Slowing PHVR n (%)	3 (10.0%)	2 (6.7) (p = 0.100)^b^	6 (20.0) (p = 0.375)^b^

BGF, background frequency; BGA background amplitude; DHVR during hyperventilation response; PHVR post hyperventilation response.

^a^Wilcoxon test compared with baseline.

^b^Cochran's Q test. baseline, FM use and post-use.﻿

Baseline BGA was of 47.69 ± 18.60 µV, whereas BGA for FM use was of 48.45 ± 17.79 µV, and post FMs use BGA was 48.08 ± 18.30 µV. There were no statistically significant differences between baseline, FM use and post FM use BGAs (P = 0.528 and P = 0.807, respectively) (Table 3). Boxplot of background Amplitude in the three phases of the study baseline, wearing FMs and post-FMs, recording showing no significant difference (Table 3).

**Figure 2:**
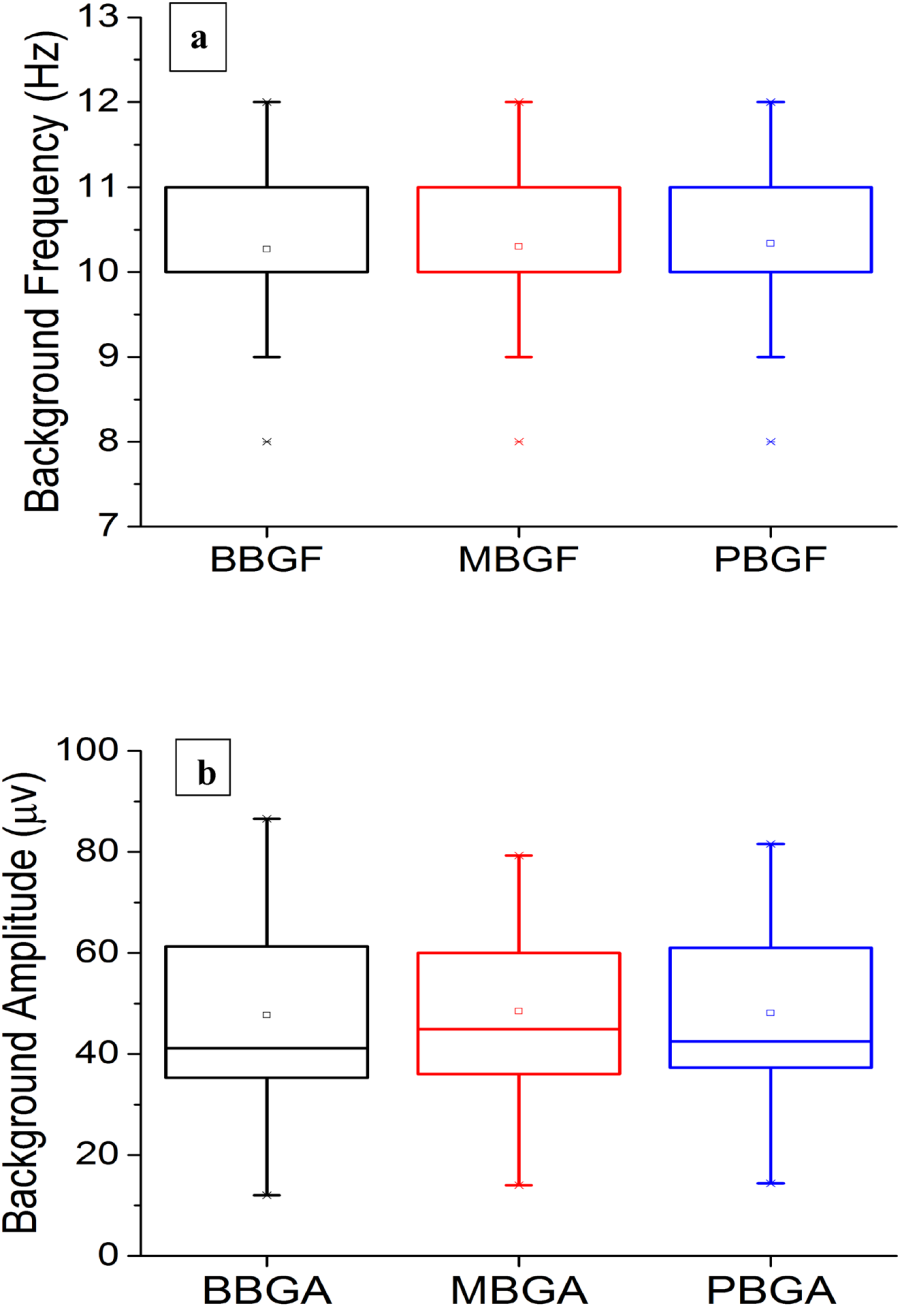
(**a**) shows a boxplot of background frequency (Hz) and (**b**) a boxplot of amplitude (µv) in the three phases of the study: baseline background, upon wearing the masks, and removal of the mask (post-mask). The record showed no significant difference in the frequency or amplitude of background rhythm between groups, with an almost identical median for the frequency and amplitude.

EEG analysis During Hyperventilation (DHVR) revealed slowing in 6 volunteers (20%) at baseline, 3(10%) during FM use and 7 (23%) post FM use 7 (22.3%). There were no statistically significant differences between baseline, FM use, and post FM DHVR; (P = 0.375 and P = 1.000), respectively (Table 3).The PHVR analyses revealed slowing in 3 volunteers (10%) at baseline, 2 (6.7%) during FM use, and 6 (20.0%) post FM use, with a total of 7 (23.3%) participants showing any PHVR slowing during the different phases, There were no statistically significant differences between baseline, FM use, and post FMs PHVR; (P = 1.000) and (P = 0.375) respectively (Table 3). No significant differences were found in the frequency and amplitude of background rhythm between the different groups [i.e. baseline background (BBG), mask background (MBG), and post-mask (PBG)] (Fig. 2a,b). Based in Wilcoxon test compared with baseline (Fig. 2a,b).

### Venous blood gases data (VBGs)

VBGs analyses revealed a PH of 7.34 ± 0.03 at baseline and 7.36 ± 0.04 during FM use. (P value = 0.362). The CO_2_ was of 47.45 ± 6.36 mmHg at baseline, 44.92 ± 10.71 during FM use (P value = 0.223). The cHO_3_P was of 25.34 ± 2.15 PmmolL at baseline, 25.03 ± 5.69 during FM use (P value = 0.762). The ctCO_2_ was 26.80 ± 2.34 PmmolL at baseline and 26.40 ± 5.98 during FM use (P value = 0.716). The BE was -0.77 ± 1.46 mmo lL at baseline and 0.05 ± 2.94 during FM use, (P value = 0.121). The SO_2_ was of 53.97 ± 19/14 at baseline and 51.79 ± 25.48 during FM use (P value = 0.714). And the O_2_ was 32.81 ± 12.67 mmHg at baseline and 29.93 ± 15.38 during FM use (P value = 0.405) No significant differences were found between baseline and post FMs in VBGs (P = 0.12–0.76) (Table 4).

**Table 4:** Venous blood gases (VBGs): baseline vs. facemask wearing and peripheral oxygen saturation (SpO_2)_ under facemask wearing.

Venous blood gases values (no = 30) cases	Baseline	FM-use	P value
pH	7.34 ± 0.03	7.36 ± 0.04	0.362
CO_2_ mmHg	47.45 ± 6.36	44.92 ± 10.71	0.223
cHCO_3_ mmoIL	25.34 ± 2.15	25.03 ± 5.69	0.762
ctCO_2_P mmoIL	26.80 ± 2.34	26.40 ± 5.98	0.716
BE mmoIL	− 0.77 ± 1.46	0.05 ± 2.94	0.121
SO_2_ mmHg	53.97 ± 19.14	51.79 ± 25.48	0.714
PO_2_ mmHg	32.81 ± 12.67	29.93 ± 15.38	0.405
SpO_2_%	–	98.19 ± 1.6%	–

FM, facemask; pH, potential hydrogen; CO_2_, carbon dioxide; cHCO_3_, concentration of hydrogen carbonate; ctCO_2_, total carbon dioxide concentration in plasma; BE, base excess; SO_2,_ oxygen saturation; SpO_2,_ peripheral oxygen saturation.

Differences between dependent variables were analyzed using Student’s T test for paired samples or Wilcoxon test.

Peripheral oxygen saturation measurements (SpO_2_) during FM use was normal in all volunteers oscillating between 95 and 100% (98.19 ± 1.16%) (Table 4).

## Discussion

The first recorded evidence of the use of FMs to prevent the spread of a pandemic was during the Spanish flu pandemic between 1917 and 1918^16^. Its use was subsequently extended to the operating rooms in Germany and USA during the 1920s, especially for minor surgical procedures^17^. In the 1940s, reusable gauze FMs gained popularity in surgical settings^17,18^. During the 1960s, the use of disposable FMs was introduced in the USA. This new type of FM was also used to protect healthcare workers from airborne infections by blocking the transmission of bacteria or viruses through respiratory droplets^19^. The use of disposable FMs shortly spread to the rest of the world.

During the current pandemic there has been great debate over the use and effectiveness of FMs to prevent the propagation of the COVID-19 virus. Research has shown that the use of medical FMs was able to reduce the transmission of the virus by up to 85%^5^. However, the use of FMs is also associated with some disadvantages involving the emotional function and social interactions. For example, FM use may interfere with facial expressions such, anger, disgust, fear, happiness, or sadness^19^. In addition, use of FMs may hinder communication as it may alter the tone and quality of the voice^19,20^. It has also been reported that FM use could lead to an impaired immune response, altered cardio-metabolic stress response, renal function, brain metabolism and mental health^21^. Rosner et al. has reported that the prolonged use of masks results in a host of physiologic and psychologic burdens and could decrease work efficiency^14^. Furthermore, it could have a negative impact on the cardiopulmonary capacity mainly during physical effort^22^, potentially leading to sudden death during heavy exercise^23^. However, a recent study^24^ performed on 11 young and healthy subjects showed that exercise under FM use does not present a physiological barrier to physical exercise, from a cardiovascular and oxygenation perspective. In our study of we observed that SpO_2_, remained within the normal range after FM use for a period of 70 min in resting conditions.

Previous studies have shown that FMs, can exert a significant respiratory effect, through CO_2_ rebreathing due to CO_2_ accumulation under the face mask, and decreased inhaled the O_2_ concentration. Resulting in hypercapnia which may lead to headaches, sweating and dizziness^18^. Although similar symptoms are also associated with orthostatic intolerance, often present in cases of hypocapnia^25^. Moreover, some studies have shown that FM use could also cause hypocapnia in addition to hypercapnia^25^. However, in our study, no VBGs changes were observed at the different stages of FM use.

Previous research has found that the use of FM was associated with a significant change in baseline bold levels in gray matter on a fMRI study^15^. Moreover, Law et al. observed that the end-tidal CO_2_ capnography, increased by an average of 7.4% induced by FM use^15^. However, this report has several limitations mentioned by other authors^26^. Accordingly, Scholkmann et al., highlighted that the end-tidal CO_2_ is not an optimal marker to assess hypercapnia induced by FM use, and suggested that a simultaneous monitoring of brain activity and hemodynamics would be needed to evaluate the relation between FM use and brain activity^26^. Our findings are similar to the results of previous research in which changes in the hemodynamic conditions of the brain in young adults who used FMs for one hour were comparable to those during daily life activities^16^. Moreover, a novelty of our study is that brain activity was monitored simultaneously with VBGs (i.e., at baseline, during FM use and post FM use), with no significant changes between the different measurement points. These results are in agreement with previous reports in which FM use was not associated with VBGs changes^22,27,28^.

Electroencephalography (EEG) offers a continuous, real time, non invasive measures of brain function^17^. However, to the best of our knowledge, our study is the first to analyze the effect of FM use on the neuro-electrical activity of the brain. In our study, there was no association between FM use and EEG changes during normal ventilation, hyperventilation and post hyperventilation. This may not be surprising considering that we did not find any increase in blood CO_2_ level, which could lead to decreased EEG activation and impaired mental and psychomotor function^29^. Our study is susceptible to certain limitations. It only investigated the short term effect of FM use and did not include older or unhealthy individuals. Therefore, these results cannot be extrapolated to older age groups and to patients with respiratory and cardiac conditions or cerebral disorders like stroke and epilepsy. Larger studies are needed to analyze the effect of a prolonged use of FMs on the respiratory and brain functions.

Furthermore, we only chose BGF and BGA of EEG as the measurements of brain neuro-electrical activity. These measures may not be as sensitive as other biomarkers, such as genetic, biochemical and neuroimaging biomarkers to assess the brain neuro-electrical activity. These latter biomarkers need to be tested to characterize the effect of surgical FM on brain neuro-electrical activity. In this respect, it is noteworthy that the current exploratory study using EEG as simple, practical neurophysiological biomarker was carried out under the extreme situation of complete lockdown in the country with restricted use of medical resources and where the hospital service was limited to emergency cases and care of COVID 19 patients.

## Conclusions

Short-term use of FM in young healthy individuals has no significant impact on the brain's neuro-electrical activity, blood venous gases or oxygen level. Moreover, no evidence of FM associated hypoxemia or hypercapnia were observed. Further studies will be needed to determine the impact of FM use in older age groups and in patients with chronic illnesses as well as the use of other biomarkers, such as genetic, biochemical and neuroimaging biomarkers to assess the brain neuro-electrical activity.

## Data Availability

All data and material are available in the office of corresponding author.

## Abbreviations

SARS: Acute respiratory syndrome
BGA: Background amplitude
BGF: Background frequency
cCHO_3_: Concentration of hydrogen bicarbonate
BE: Base excess
CO_2_: Carbon dioxide
CBF: Cerebral blood flow
COVID-19: Coronavirus disease 2019
DHVR: During hyperventilation response
EEG: Electroencephalograph
ET: End-tidal
FMs: Facemasks
EFV1: Forced expiratory volume in one second
FVC: Forced vital capacity
HV: Hyperventilation
IRB: Institutional Review Board
JUH: Jordan University Hospital
O_2_: Oxygen
SO_2_: Oxygen saturation
PRC: People’s Republic of China
SpO_2_: Peripheral oxygen saturation
pH: Potential hydrogen
PHVR: Post hyperventilation response
SD: Standard deviation
ctCO_2_P: Total carbon dioxide concentration in plasma
VBGs: Venous blood gases
WHO: World Health Organization
